# The Ribosomal Biogenesis Protein Utp21 Interacts with Hsp90 and Has Differing Requirements for Hsp90-Associated Proteins

**DOI:** 10.1371/journal.pone.0092569

**Published:** 2014-03-19

**Authors:** Victoria R. Tenge, Jared Knowles, Jill L. Johnson

**Affiliations:** Department of Biological Sciences and the Center for Reproductive Biology, University of Idaho, Moscow, Idaho, United States of America; University of Pittsburgh, United States of America

## Abstract

The molecular chaperone Hsp90 buffers the effects of genetic variation by assisting the stabilization and folding of multiple clients critical for cell signaling and growth. We identified an interaction of Hsp90 and associated proteins with the essential nucleolar protein, Utp21, part of a large complex required for biogenesis of the small ribosomal subunit. The *utp21-S602F* mutation, which causes minor defects in otherwise wild-type yeast, exhibited severe or lethal growth defects when combined with mutations in Hsp90 or co-chaperones. WT Utp21 and Utp21-S602F exhibited similar interactions with Hsp90, and steady-state levels of WT Utp21 were reduced upon Hsp90 mutation or inhibition. Mutations in the human homolog of *UTP21, WDR36,* have been associated with adult-onset primary open-angle glaucoma, a leading cause of blindness worldwide. Three different mutant forms of Utp21 analogous to glaucoma-associated *WDR36* mutations exhibit reduced levels in yeast cells expressing mutations in Hsp90 or associated chaperones, suggesting that Hsp90 and co-chaperones buffer the effects of those mutations.

## Introduction

Efficient protein folding and assembly of multiprotein complexes is critical for cell survival. The Hsp90 molecular chaperone is required for the folding and maturation of hundreds of cytosolic and nuclear proteins that play key roles in cellular signaling pathways [1], [2]. The interaction of Hsp90 with client proteins requires additional proteins, including the Hsp70 and Hsp40 molecular chaperones, which bind clients prior to Hsp90. Hsp90 function is also dependent on co-chaperones that directly bind Hsp90 and have diverse roles such as regulating the ATPase activity or conformational changes of Hsp90 or promoting Hsp90-client interaction. The Hsp90 co-chaperone Sti1 (Hop in mammalian cells) is a key component of the Hsp90 folding pathway. Sti1 interacts directly with both Hsp70 and Hsp90, and plays a role in the transfer of clients from Hsp70 to Hsp90 for folding [3], [4].

In order to identify proteins that require Sti1 for folding, we conducted a *STI1* synthetic lethal screen in *Saccharomyces cerevisiae* [5]. We isolated a mutation in the essential gene *UTP21*, *utp21-S602F*. A genome-wide analysis of Hsp90 function also identified a connection between Hsp90 and Utp21 [6]. Utp21 (U three protein 21) localizes to the nucleolus and is part of a large (2.2-MDa, 80S) ribonucleoprotein complex required for the processing, assembly and maturation of the 40S ribosome [7], [8]. Most Utps are essential, and genetic depletion of many of these components results in loss of the 18S rRNA, which is derived from the 35S pre-RNA [9]. Mutations in the human homolog of *UTP21, WDR36,* have been associated with adult-onset primary open-angle glaucoma (POAG), a leading cause of blindness worldwide [10]. A subset of *UTP21* mutations analogous to glaucoma-associated mutations in *WDR36* also exhibited enhanced growth defects when combined with a deletion in *STI1* [11], suggesting Sti1 has a general role in regulating Utp21 function.

Hop, the mammalian homolog of Sti1, localizes to both the cytoplasm and nucleus [12], but no nucleolar-specific functions of Hop or Sti1 have been described. In *S. cerevisiae*, Sti1 is required for the folding and activation of many Hsp90 clients, including the native Ste11 kinase as well as heterologous glucocorticoid receptor and v-src kinase [13], [14]. Sti1 has also been shown to play a role in the degradation of the VHL tumor suppressor [15]. Given the established functions of Hsp90 in protein folding and assembly of protein complexes [16], we determined whether Utp21 interacts with Hsp90 and whether Utp21 stability is dependent on Hsp90. We also examined the impact of mutations in other components of the Hsp90 molecular chaperone on WT and mutant Utp21. Finally we examined whether glaucoma-associated mutations in Utp21 affect chaperone dependence. Our results confirm a role for Hsp90 and associated proteins in the folding and stability of Utp21.

## Results

Utp21 is an essential 939 amino acid nucleolar protein that is part of a large complex required for 18S ribosomal RNA biogenesis [7], [9], [17]. It contains multiple WD40 repeats predicted to form a double seven-bladed β-propeller structure. The *utp21-S602F* mutation causes minor growth defects in otherwise WT cells, but a severe or lethal growth defect in the absence of *STI1* [5], [11]. Although Sti1 is known to transfer misfolded client proteins from Hsp70 to Hsp90 for folding [4], the role of Sti1 in Utp21 function is poorly understood, and the role of Hsp90 in Utp21 folding and/or function has not been established.

### Utp21 Genetically Interacts with Hsp90, Hsp70, Sti1, Sse1 and Ydj1

**Figure 1:**
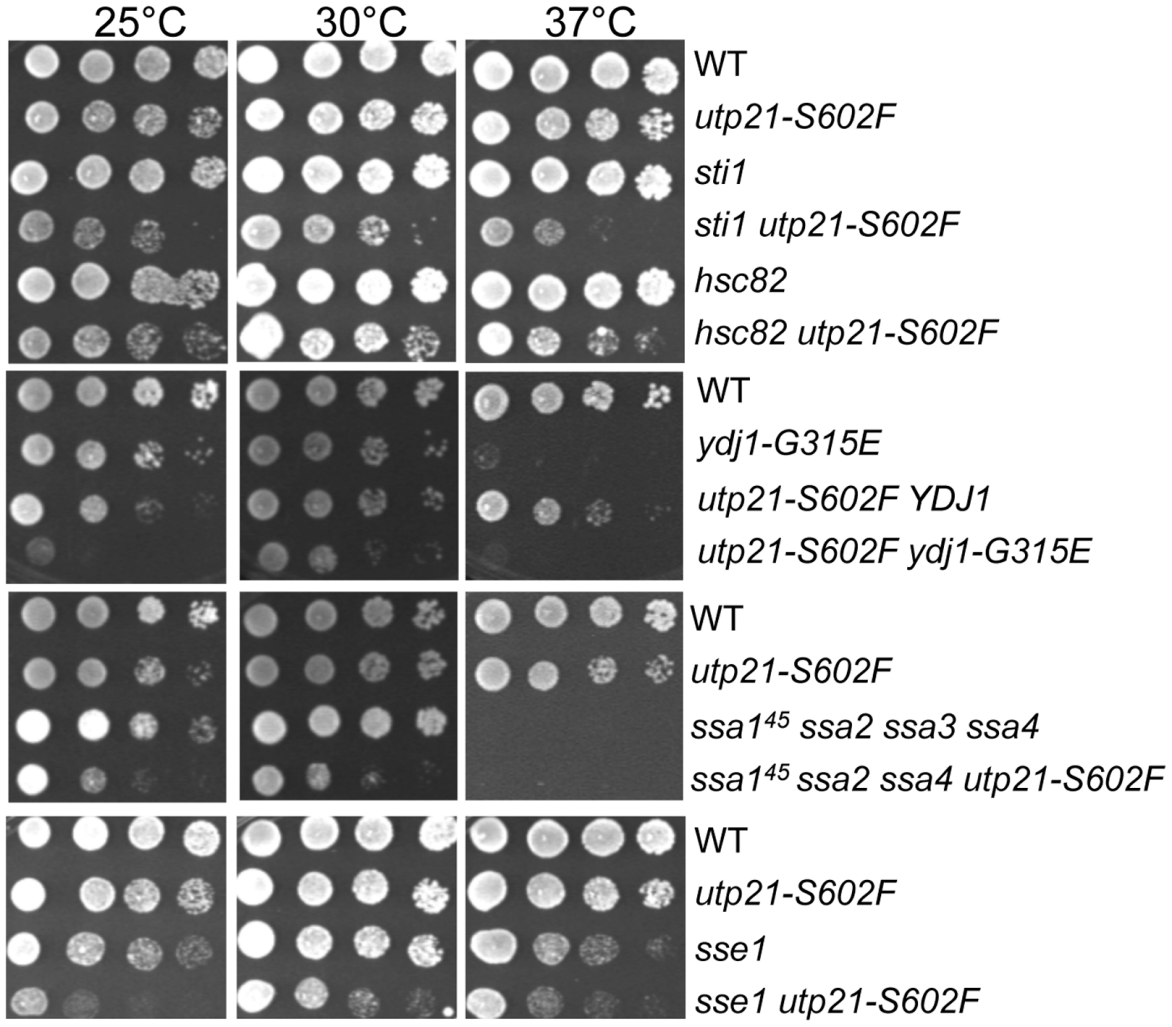
Cells expressing *utp21-S602F* require Sti1, Hsp90, Hsp70, Hsp40 and Sse1. Yeast strains expressing WT *UTP21* or *utp21-S602F* in combination with the indicated chaperone mutation were grown overnight, serially diluted 10-fold and grown for 2 days at the indicated temperature.

In yeast, Hsp90 is encoded by two genes, *HSC82* and *HSP82*, one of which must be present for viability [18]. We determined whether strains containing a deletion of *HSC82* exhibit enhanced growth defects in cells containing the *utp21-S602F* mutation. As shown in Figure 1, the *utp21-S602F* mutation or *STI1* deletion causes only mild growth defect at 25°C and 37°C. However, cells that lack *STI1* and contain the *utp21-S602F* mutation exhibit slow, temperature-sensitive growth at all three temperatures. Similar synthetic growth defects were observed in *utp21-S602F* cells that lack *HSC82*. Prior studies have also observed synthetic growth defects upon *HSC82* deletion despite the presence of the heat inducible *HSP82* [19].

Hsp90 interacts with clients that have some secondary and tertiary structure [20], [21], necessitating prior client interaction with the molecular chaperones Hsp70 and Hsp40 [22], which preferentially interact with unfolded proteins [23]. Hsp70s of the Ssa family (Ssa1-4), the Hsp40 Ydj1 and the Hsp70 nucleotide exchange factor Sse1 physically or functionally interact with Hsp90 clients [16], [24], [25]. Strains expressing *ydj1-G315E* or the *ssa1^45^* allele in conjunction with deletion of *SSA2, SSA3* and *SSA4* exhibit near wild-type growth at 23°C and 30°C, but severe growth defects at 37°C [26], [27]. As shown in Figure 1, combination of the *utp21-S602F* mutation with *ydj1-G315E* or the *ssa1^45^* mutation in conjunction with deletion of *SSA2* and *SSA4* resulted in strong growth defects at both 25°C and 30°C. Likewise, cells expressing *utp21-S602F* in combination with a deletion of *SSE1* exhibited slow growth phenotypes at all three temperatures. Together, these results indicate that Utp21-S602F folding, stability and/or function are dependent on Sti1, Hsp90, Ydj1, Ssa and Sse1.

**Figure 2:**
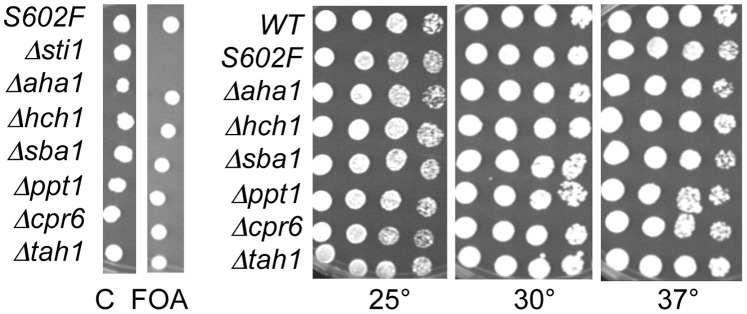
No synthetic growth defects were observed in additional Hsp90 co-chaperone deletion strains. **A.** Strains contained the *utp21-S602F* mutation, a chromosomal deletion of the indicated co-chaperone, and a pRS316*ADE3* plasmid expressing the deleted co-chaperone. Synthetic lethality was assessed by spotting cells on 5-FOA or uracil drop-out plates as a control (C). Plates were incubated for two days at 30°C. **B.** Cells that arose on 5-FOA were grown overnight, serially diluted 10-fold, and grown for two days at the indicated temperature. All strains except WT contained the *utp21-S602F* mutation.

*S. cerevisiae* contains at least ten Hsp90 co-chaperones in addition to Sti1 [3]. We constructed strains similar to the original isolate of *utp21-S602F* (*sti1 utp21-S602F/pRS316ADE3STI1*) that contained a chromosomal deletion of the gene encoding the indicated co-chaperone as well as a plasmid expressing that co-chaperone. Only deletion of *STI1* resulted in severe growth defects in the presence of 5-FOA, which counterselects for the pRS316-co-chaperone plasmid (Figure 2A). We further monitored growth of cells that contain the *utp21-S602F* mutation in combination with deletion of the indicated co-chaperone. No significant growth defects were observed upon deletion of any other co-chaperones (Figure 2B). Folding of the well-established Hsp90 client, the progesterone receptor, is known to proceed from early complexes consisting of Hsp70, Ydj1, Sti1/Hop and Sse1 to late complexes consisting of other cochaperones such as p23/Sba1, FKBP51/52 and Cyp40/Cpr6 [28], [29]. Our results indicate that Utp21-S602F is specifically dependent on proteins that function early, but not late, in the Hsp90 folding pathway.

### WT Utp21 and Utp21-S602F are Expressed at Similar Steady-state Levels

**Figure 3:**
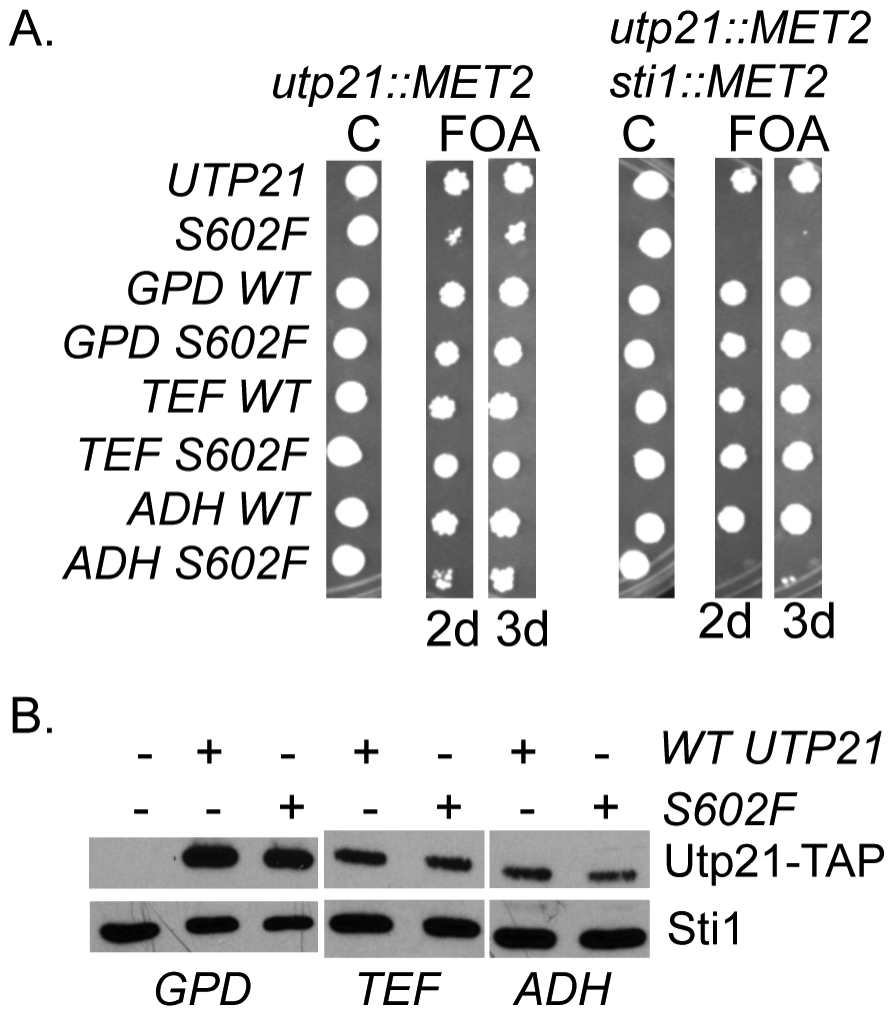
WT Utp21 and Utp21-S602F are expressed at similar steady-state levels. **A.** WT or mutant *UTP21* expressed under the endogenous promoter or WT or mutant Utp21-TAP expressed under the *GPD*, *TEF* or *ADH* promoter was transformed into JJ666 (*utp21::MET2/*YcP50-*UTP21*) or JJ600 (*sti1::MET2 utp21::MET2*/YCp50-*UTP21*). Synthetic lethality was assessed by spotting cells on 5-FOA or uracil drop-out plates as a control (C). Plates were incubated for two or three days at 30°C as indicated. **B.** Whole cell extracts from indicated cells were separated by SDS-PAGE and immunoblotted with anti-TAP antibodies or anti-Sti1 antibodies as a loading control.

Prior analyses did not examine the effect of Utp21 mutation on its steady-state level in the cell. We established a system to monitor the effect of chaperone mutation on Utp21 levels and Utp21-Hsp90 interaction. In order to avoid possible effects of Utp21 overexpression, we identified a plasmid-borne version of Utp21-S602F-TAP that exhibited growth defects similar to endogenous *utp21-S602F.* We transformed plasmids expressing WT *UTP21* or *upt21-S602F* under the endogenous promoter or promoters that allow for varying levels of constitutive expression [30] into WT or *sti1* cells containing a chromosomal deletion of *UTP21*. Transformants were grown overnight in selective media then spotted onto plates containing 5-FOA or uracil dropout plates as a control (Figure 3A). As expected, no growth was observed when *sti1 utp21* cells expressing *utp21-S602F* from its own promoter were grown in the presence of 5-FOA for two days, but small colonies appeared after three days. Similar growth defects were observed when *utp21-S602F* was expressed from the *ADH* promoter. As expected, the level of Utp21-TAP expression was highest when expressed under the *GPD* promoter and lowest when expressed under the *ADH* promoter. However, with all promoters tested, WT and mutant Utp21 were expressed at similar levels (Figure 3B). The lack of a *STI1* synthetic phenotype in cells expressing *utp21-S602F* under the *TEF* and *GPD* promoters suggests that *utp21-S602F* mutation causes a loss of function that may be overcome by overexpression.

### The Combination of *utp21-S602F* and Mutant Hsp90 is Lethal

**Figure 4:**
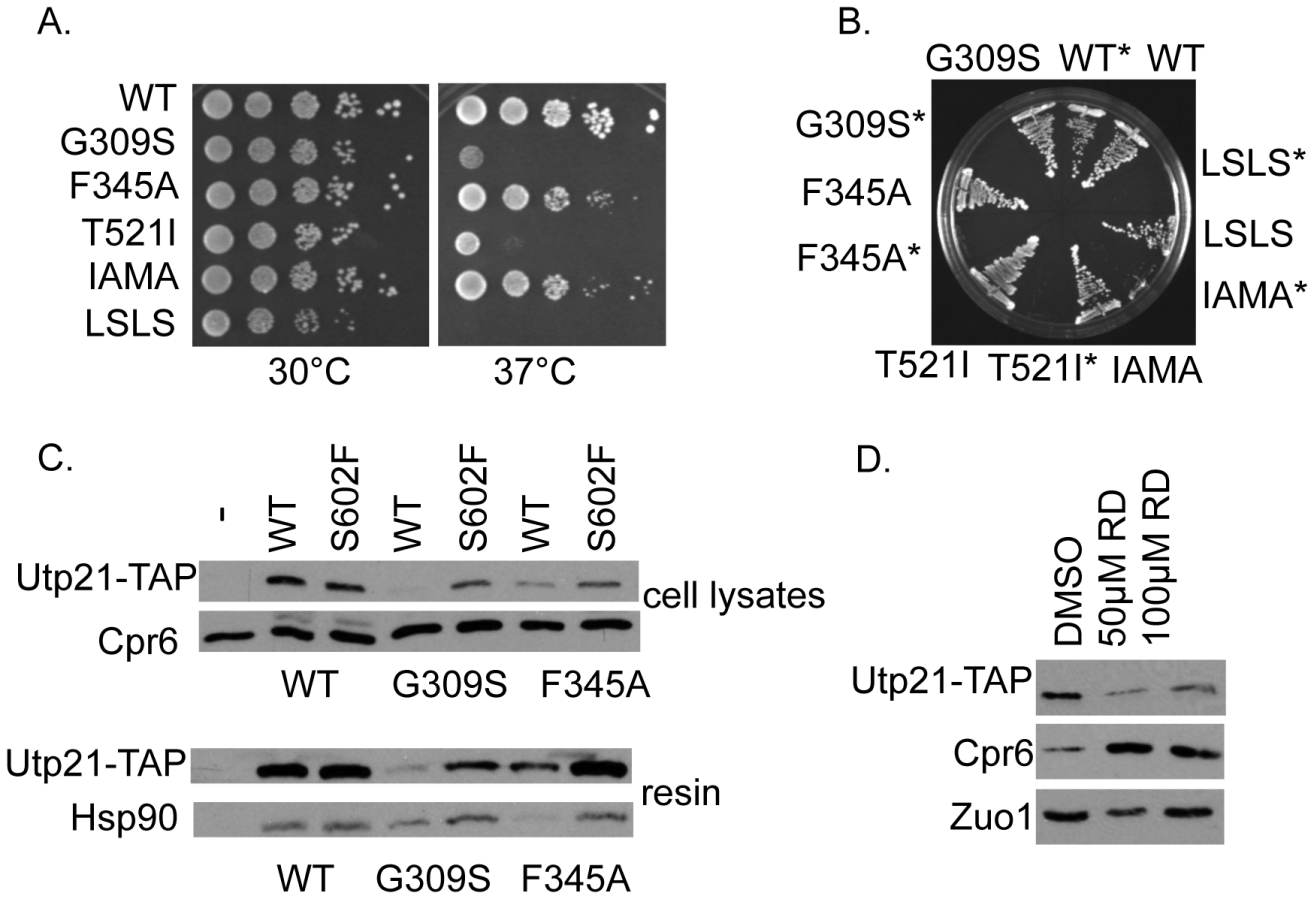
Effect of Hsp90 mutation or inhibition on Utp21. **A.** Plasmids expressing WT or mutant Hsp90 were transformed into strain JJ816 (*hsc82 hsp82/*YEp24-*HSP82).* Transformants were struck out onto media containing 5-FOA to counterselect for the plasmid expressing WT *HSP82*. Resultant colonies were serially diluted 10-fold and assayed for growth after 2 days at the indicated temperature. **B.** Plasmids expressing WT or mutant Hsp90 were transformed into *hsc82 hsp82* or *hsc82 hsp82 utp21-S602F* cells (marked with an asterisk *), then grown on 5-FOA plates for three days at 30°C. Hsp90-I588AM589A was abbreviated as IAMA and Hsp90-L647SL648S was abbreviated as LSLS. **C.** Strain JJ591 expressing untagged WT Utp21 or expressing WT or mutant ADH-Utp21-TAP was transformed with plasmids expressing WT or mutant Hsp90. Utp21-TAP complexes were isolated with IgG sepharose and analyzed by SDS-PAGE and immunoblot analysis. **D.** Strain JJ666 expressing ADH-Utp21-TAP was grown overnight in rich media prior to dilution into fresh media. DMSO (vehicle) or radicicol at the indicated final concentration was added to cells in exponential growth. After four hours, cells were harvested and analyzed by SDS-PAGE and immunoblot analysis.

Amino acid mutations throughout Hsp90 cause defects in client activity and or ATP hydrolysis [31]–[33]. We analyzed the combined effect of the *utp21-S602F* mutation and a set of temperature sensitive mutations that alter residues located in either the middle or the carboxy-terminal domain of Hsp90 (the Hsc82 isoform) (Figure 4A) [34], [35]. Although all of the mutants exhibit near WT growth at 30°C in the presence of WT *UTP21* (Figure 4A), all of the Hsp90 mutants were inviable at 30°C when combined with the *utp21-S602F* mutation (Figure 4B).

Many Hsp90 clients become unstable upon Hsp90 inhibition and are subsequently degraded by the proteosome [36], [37]. We determined the impact of Hsp90 mutation on Utp21-TAP levels and Utp21-Hsp90 interaction. This was possible since growth defects of cells expressing Utp21-S602F-TAP under the *ADH* promoter were less severe relative to cells expressing *utp21-S602F* from the endogenous chromosomal location (not shown). WT or mutant Utp21-TAP was isolated from cells under non-denaturing conditions. The level of WT Utp21 in cell lysates was reduced in cells expressing *hsp90-G309S* or *-F345A* (Figure 4C, top panels). The level of Utp21-S602F was also reduced in these strains, but to a lesser extent. As shown in Figure 4C bottom panels, WT Utp21 and S602F bound similar levels of WT Hsp90. Neither of the Hsp90 mutants we tested abolished Utp21 interaction. Since the steady-state levels of WT Utp21-TAP were greatly reduced in *hsp90-G309S* cells, we examined the effect of the Hsp90 inhibitor radicicol (Figure 4D). Exponentially growing yeast were treated with vehicle alone (DMSO), 50 μM radicicol or 100 μM radicicol. Treatment with radicicol for four hours resulted in reduced Utp21-TAP levels, indicating that Utp21 stability is dependent on Hsp90. Since Hsp90 inhibition results in activation of the heat shock response through activation of Heat Shock Factor (HSF) [38], we confirmed that radicicol treatment resulted in increased levels of the Hsp90 co-chaperone Cpr6, which is known to be regulated by HSF. For comparison, the level of Zuo1, a chaperone that is not regulated by HSF, was unaffected by treatment with the drug [39].

### Other Mutant Forms of Utp21 are also Sensitive to Hsp90 Mutation

**Figure 5:**
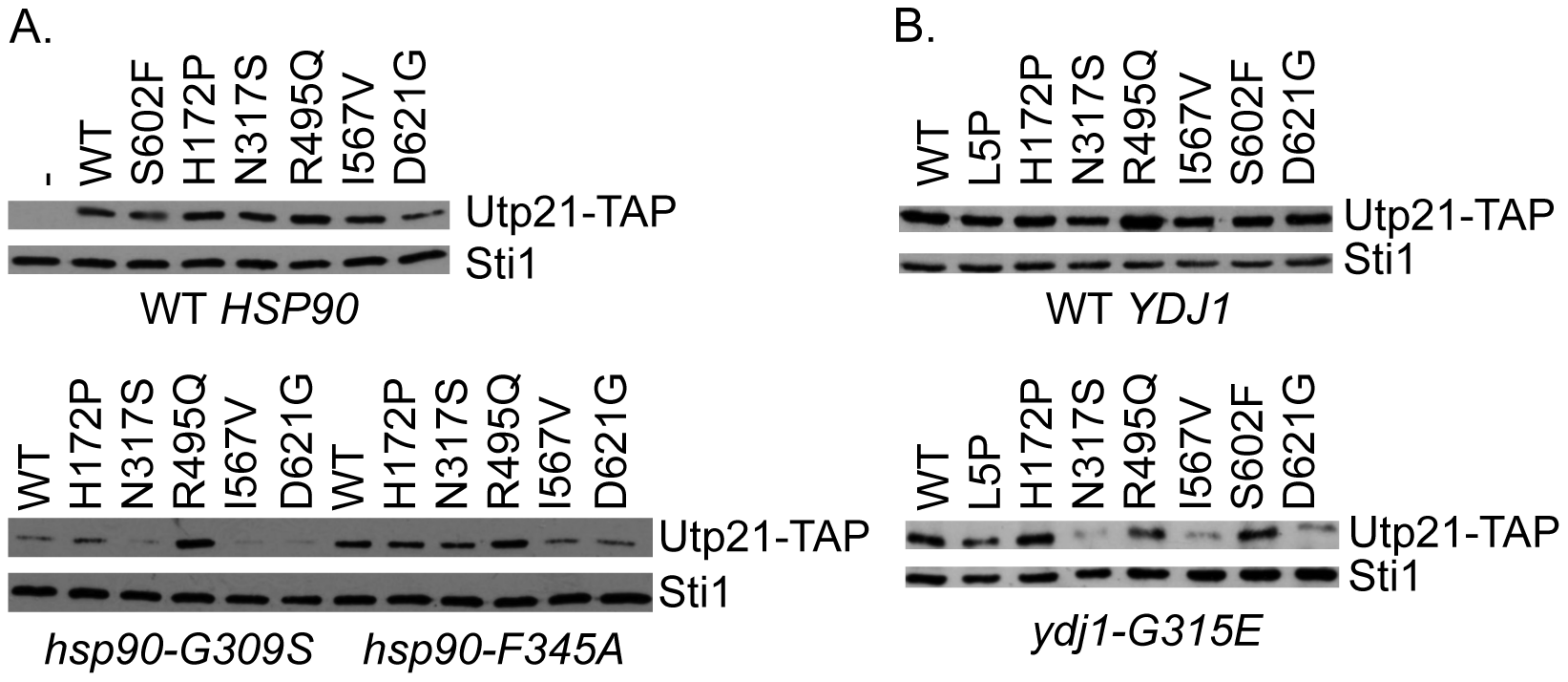
Effect of Hsp90 or Ydj1 mutation on additional Utp21 mutants. **A.** Plasmids expressing WT or mutant ADH-Utp21-TAP were transformed into strain JJ591 expressing WT (upper panel) or mutant Hsp90 (lower panel). **B.** Plasmids expressing WT or mutant ADH-Utp21-TAP were transformed into strain JJ160 expressing WT *YDJ1* (upper) or *ydj1-G315E* (lower). Whole cell lysates were analyzed by SDS-PAGE and immunoblot analysis.

Mutations in the human homolog of *UTP21*, *WDR36*, have been associated with glaucoma [10]. Footz et al. introduced mutations analogous to glaucoma-associated alleles into *UTP21* [11]. Similar to *utp21-S602F,* four of the eleven mutants tested exhibited enhanced growth defects in the absence of *STI1*. We constructed TAP-tagged versions of five of those mutants, three that showed *STI1*-dependent growth (R495Q, I567V and D621G) and two that did not (H172P and N317S). As shown in Figure 5A, all of the Utp21 mutants were expressed at similar levels in cells expressing WT *HSP90*. The steady-state levels of most forms of Utp21, including WT, were very low in cells expressing *hsp90-G309S*. Utp21-N317S, -I567V and D621G displayed the lowest levels. Utp21-R495Q, similar to S602F (Figure 4C), was less affected by this mutation. A different pattern was observed in cells expressing *hsp90-F345A*. Only Utp21-I567V and -D621G were significantly affected by that mutation. Unlike *utp21-S602F*, none of these mutations resulted in enhanced growth defects when expressed in cells expressing mutations in Hsp90 (not shown). Loss of *STI1* did not affect the level of WT or mutant Utp21-TAP (not shown). However, *YDJ1* mutation did affect the levels of some mutants. As shown in Figure 5B, Utp21-N317S, -I567V and -D621G displayed reduced levels in cells expressing *ydj1-G315E*. Utp21-L5P is another mutant that did not exhibit enhanced growth defects in cells lacking *STI1* [11].

### Utp21-S602F-GFP Exhibits Altered Localization

**Figure 6:**
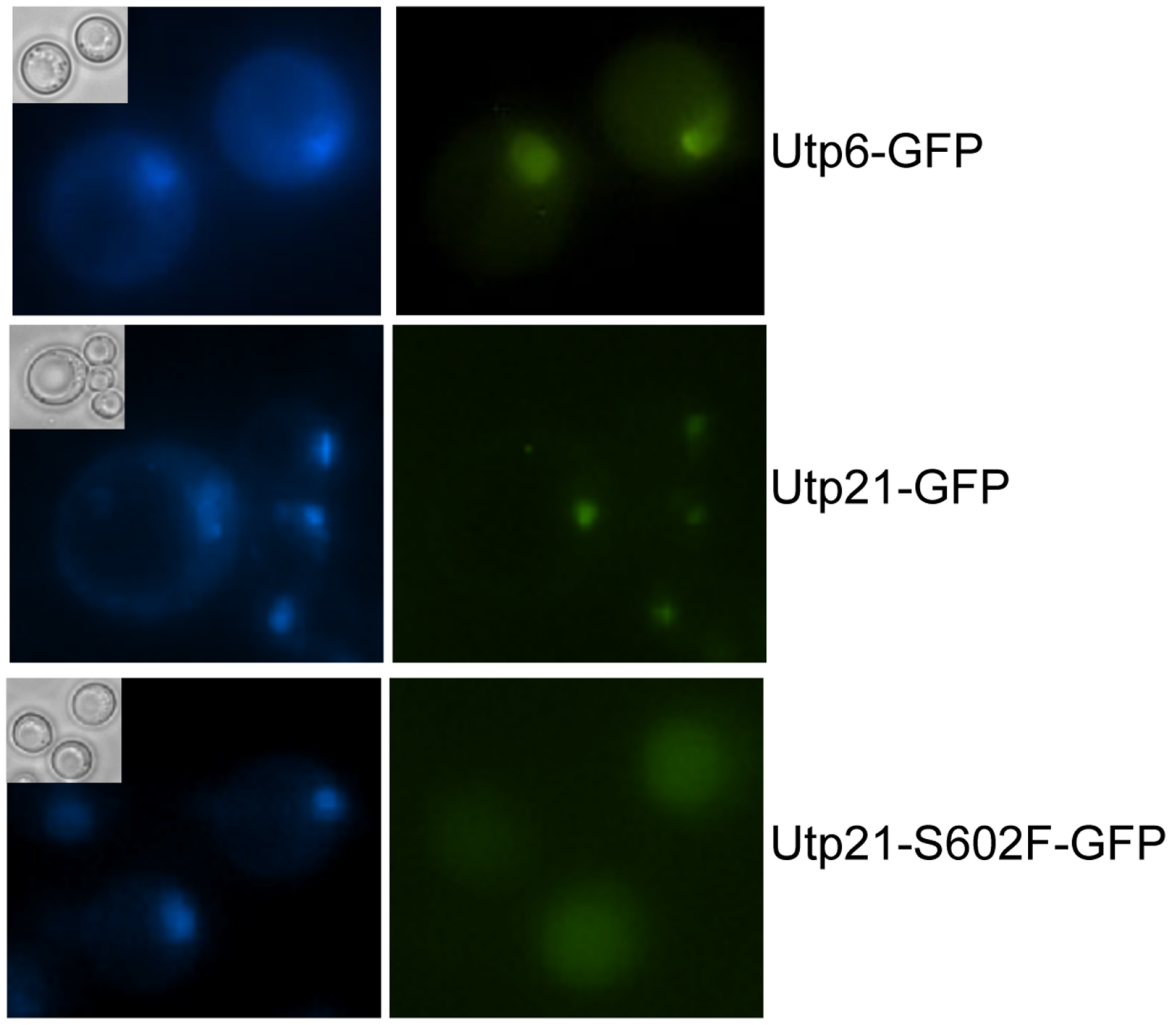
Utp21-S602F does not exhibit strong nucleolar staining. **A.** Cells expressing Utp21-GFP, Utp21-S602F-GFP or Utp6-GFP from the normal chromosomal location were grown overnight in the presence of 10 μg/ml DAPI. Phase image (inset), DAPI (left) and GFP (right) immunofluorescence microscopy was performed on unfixed cells.

Since the combination of *utp21-S602F* and mutations in components of the Hsp90 molecular chaperone machine result in strong growth defects that cannot be fully explained by reduced levels of Utp21-S602F, we examined the effect of this mutation on localization. Utp21 and other components of the ribonuclear complex, such as Utp6, exhibit nucleolar localization [7]–[9]. To examine the effect of the *utp21-S602F* mutation on localization of Utp21, we took advantage of a commercially available strain in which Utp21-GFP was expressed from the normal chromosomal location [40]. Utp21-GFP was confirmed to co-localize with established nucleolar proteins in that study. As shown in Figure 6, both Utp21-GFP and Utp6-GFP (as a control) display foci that overlap with the DAPI staining, consistent with nucleolar staining. However, in cells expressing Utp21-S602F-GFP, the GFP staining was dispersed throughout the cell. This suggests that in otherwise WT cells, reduced levels of Utp21-S602F localize to the nucleolus, but enough is correctly localized to support essential functions. The level of WT and mutant Utp21-GFP was too low to observe with immunoblot analysis. Immunoprecipitation of WT and mutant Utp21-GFP suggested that WT Utp21-GFP and Utp21-S602F-GFP were of similar size. However, inconsistent levels of Utp21-S602F-GFP were observed, suggesting that the fusion protein may have altered stability (not shown). We were unable to test the effect of chaperone deletion on localization of Utp21-S602F-GFP due to the severe growth defects of those strains (not shown and Figure 1).

### Hsp90 Mutation does not Affect the Localization of WT Utp21-GFP

**Figure 7:**
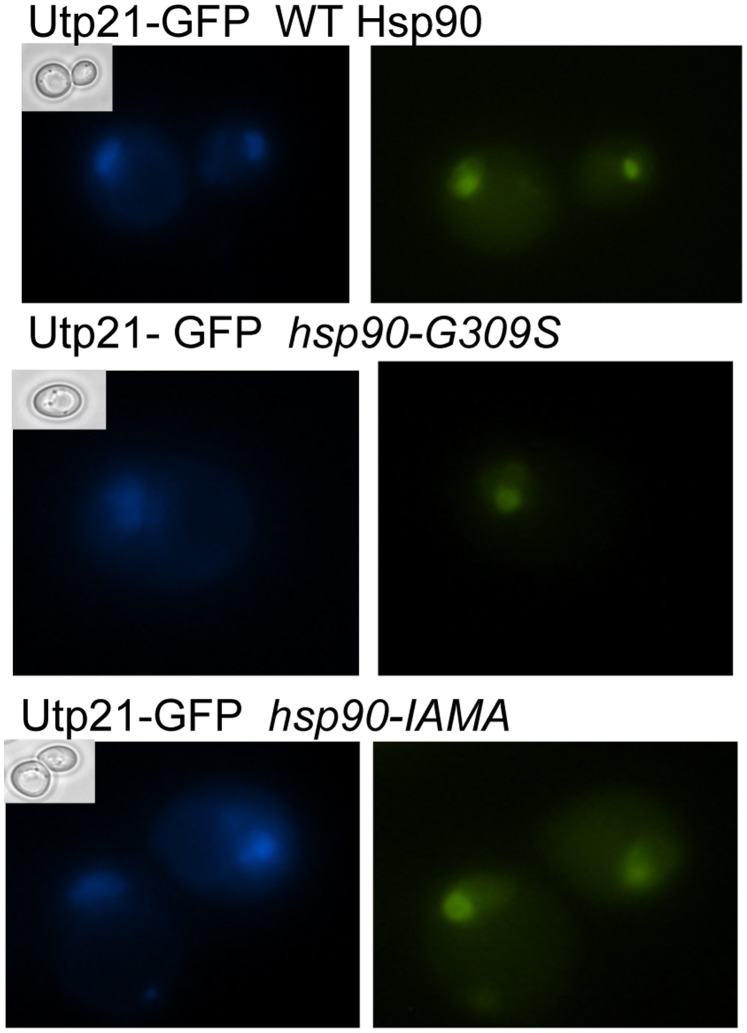
Hsp90 mutation does not disrupt the localization of WT Utp21. Strain JJ455 (*hsc82 hsp82* Utp21-GFP) was transformed with plasmids that express His-tagged WT or mutant Hsc82 and the plasmid expressing WT *HSC82* was lost by growth in the presence of 5-FOA. Cells were grown overnight in the presence of 10 μg/ml DAPI. Phase image (inset), DAPI (left) and GFP (right) immunofluorescence microscopy was performed on unfixed cells.

Hsp90 has been shown to be required for the folding, stability, activation and/or trafficking of client proteins [28]. To determine whether Hsp90 plays a role in Utp21 localization, we examined the effect of Hsp90 mutation on localization of WT Utp21. We examined Utp21-GFP localization in strains expressing WT Hsp90, *hsp90-G309S* or *hsp90-I588AM589A* (Figure 7). In each of the strains examined, a clear foci of Utp21-GFP staining was observed. Similar foci of Utp21-GFP were observed in *ssa1 ssa2 ssa3, sti1* and *sse1* strains (not shown). These results suggest that Hsp90 and co-chaperones are not required for proper localization of WT Utp21.

## Discussion

The Hsp90 molecular chaperone has previously been shown to affect the folding and activity of multiple cellular proteins, as well as playing a role in the assembly of multiprotein complexes [3], [41]. Consistent with a genome-wide study that identified Utp21 as a potential Hsp90 client [6], we identified a role of Hsp90 and associated proteins in the function of Utp21, a component of a large protein complex required for biogenesis of the small ribosomal subunit [7], [9], [17]. The combination of *utp21-S602F* and temperature-sensitive *hsp90* mutations was lethal. We also saw synthetic growth defects upon combination of *utp21-S602F* and deletion of the gene encoding constitutively expressed Hsc82, which results in lower levels of Hsp90 [18]. We found that WT Utp21 is found in complex with Hsp90, and that mutation or inhibition of Hsp90 resulted in reduced steady-state levels of WT Utp21, two hallmarks of Hsp90 clients. However, we have not yet established that Utp21 directly interacts with Hsp90 or whether Hsp90 binds another protein found in Utp21 complexes.

The Utp21-S602F mutant was genetically dependent on Hsp90, Hsp70, Ydj1, Sti1 and Sse1, early components of the Hsp90 folding cycle, but was unaffected by mutations in proteins that function later in the folding cycle [28], [29]. The reason for this difference is unclear, mainly because few studies have compared how loss of specific co-chaperones affects client folding and/or stability. It is possible that the conformation of Hsp90 is important, since early co-chaperones preferentially bind Hsp90 in the open, nucleotide-free conformation, whereas Aha1/Hch1, Cpr6 and Sba1 preferentially interact with Hsp90 in the ATP-bound conformation that occurs later in the folding cycle [3]. It is also possible that only a subset of co-chaperones is required for Utp21 folding. For example, mutations in only a subset of Hsp90 co-chaperones affect the activity of the Ste11 kinase [14], [42], and mutation in only one specific co-chaperone affected the activity of the adenylate cyclase, Cyr1 [43]. Another possibility is that the Utp21-S602F alteration specifically affects the ability of Utp21 be recognized by the quality control pathway that dictates whether proteins are folded or degraded. This hypothesis is consistent with earlier reports that suggest that components of early, but not late, Hsp90 complexes participate in client degradation [15], [29]. Although the levels of Utp21-S602F appeared less affected by Hsp90 mutation than that of WT Utp21, further experiments are necessary to determine whether Utp21-S602F exhibits altered folding and/or degradation.

Utp21-S602F and WT Utp21 levels were similar and both forms interacted with WT Hsp90. Yeast expressing the *utp21-S602F* mutation display near WT growth, yet the mutation exhibited lethality or severe growth defects when the Hsp90 molecular chaperone machine was compromised. The lethality in chaperone mutant strains is likely due to reduced levels of Utp21-S602F or enhanced misfolding defects that prevent it from performing its essential functions. Our results suggest Utp21-S602F has reduced nucleolar localization, which would prevent it from assembling with other components for efficient pre-rRNA processing, but our Utp21-S602F-GFP may have altered stability relative to Utp21-GFP. In yeast, depletion of many of the proteins required for biosynthesis of the small ribosomal subunit results in reduced levels of mature 18S rRNA, and elevated levels of pre-rRNA [7], [9], [17]. A recent report describes similar results upon depletion of the mammalian counterparts, including WDR36 [44]. Footz et al previously examined the effect of *utp21* mutations on pre-rRNA levels [11]. Cells expressing *utp21-R495Q*, *-I567V, -S602F* or *-D621G* exhibited reduced pre-RNA levels when expressed in an otherwise wild-type background. They were unable to examine the combined effect of *sti1* deletion and *utp21-S602F* in that assay due to synthetic lethality. However, the other mutants exhibited elevated levels of pre-RNA levels when combined with *sti1* deletion, suggesting that loss of *STI1* directly alters the function of Utp21 in rRNA processing. The synthetic lethality makes analysis of the effect of combination of *utp21* and *hsp90* mutations on 18S rRNA processing and biogenesis of the small ribosomal subunit difficult. Hsp90 mutations also likely have pleotropic effects on ribosome function, as they have been found to affect polysome stability [45]. We have not directly tested whether the synthetic growth defects observed upon combination of *utp21-S602F* and chaperone mutations is due to harmful effects of misfolded Utp21-S602F. Our results that overexpression of Utp21-S602F alleviates the *STI1*-synthetic growth phenotype (Figure 3), are more consistent with a loss of function phenotype of that mutant.

**Figure 8:**
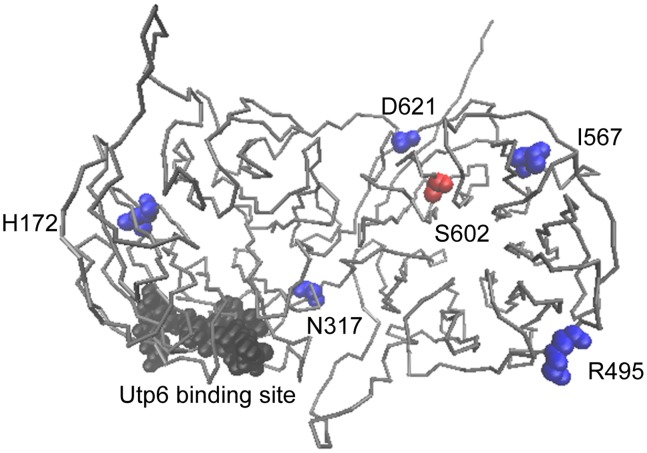
Location of mutated Utp21 amino acids in predicted structure. Model was generated using the LOMETS program from the Zhang lab (http://zhanglab.ccmb.med.umich.edu/LOMETS/) [48]. Residue S602F is shown in red. Residues homologous to mutated WDR36 residues found in glaucoma patients are indicated in blue [11]. Utp6 was previously shown to interact with a.a. 267–279 of Utp21 (shown in black) [47]. Figure generated using VMD [61].

During ribosome assembly, Utp21 is part of a smaller complex that is essential for the initial assembly steps of the 90S pre-ribosome. That complex includes Pwp2, Dip2, Utp6, Utp13 Utp18 and Utp21 [46]. A prior study established that Utp21 and Utp6 interact directly using a yeast two-hybrid assay [47]. Although the structure of Utp21 is unknown, we generated a model based on homology with other proteins [48]. All of the mutations we examined are predicted to be within the WD40 motifs implicated in protein-protein interactions, but none of the mutations we examined are located in the Utp6 interaction site (Figure 8). Additional studies are required to determine whether the Utp21-S602F alteration disrupts interaction with Pwp2, Dip2, Utp6, Utp13 Utp18 or other proteins required for ribosomal assembly. Based on our results and those of others [11], the I567V and D621G mutations result in a shared set of defects: *STI1*-dependent growth, elevated pre-rRNA levels and reduced levels in Hsp90 and Ydj1 mutant cells. Consistent with the shared phenotypes, the I567 and D621 residues appear closest to S602 in the predicted structure (Figure 8). The R495Q and N317S alterations affect a subset of these functions. R495Q caused *STI1*-dependent growth and elevated levels in *hsp90-G309S* cells, suggesting it may cause defects similar to those observed with S602F. The N317S alteration, which did not cause *STI1*-dependent growth, displayed reduced levels in cells expressing mutations in either Hsp90 and/or Ydj1. We have not observed any chaperone-related defects of Utp21-H172P.

A clear link between *WDR36* mutation and pathogenesis remains unclear [10], [49]–[51]. However, in at least one study, the presence of *WDR36* variants was associated with more severe disease, suggesting it is a glaucoma-modifier gene [52]. The functional connection between WDR36 and Sti1 appears to be conserved in mammalian cells [53], and both Utp21 and WDR36 localize to the nucleolus and play an essential role in ribosome biogenesis [8], [44], [54]. Our results suggest that alteration of Utp21/WDR36 may result in folding defects that cause enhanced reliance on the Hsp90 molecular chaperone machinery and/or reduced nucleolar localization. Many forms of glaucoma are progressive aging diseases [55], and recent studies suggest that as cells age their ability to effectively maintain protein homeostasis declines [56]. Further studies will be needed to determine whether the corresponding mutants in WDR36 exhibit enhanced sensitivity to inhibition of Hsp90 in mammalian cells and whether this is enhanced during aging. If so, this could suggest that failure of Hsp90 to buffer the effects of *WDR36* mutation as cells age may contribute to disease.

## Materials and Methods

### Yeast Strains and Growth Assays

All yeast strains are isogeneic to W303, but contain the additional *met2* and *lys2* mutations [5], [57] (Table 1). Yeast cells were grown in either yeast extract-peptone-dextrose (YPD- 1% Bacto yeast extract, 2% peptone, and 2% dextrose) or defined synthetic complete media supplemented with 2% dextrose and were transformed by lithium acetate methods. Growth was examined by spotting 10-fold serial dilutions of yeast cultures on appropriate media, followed by incubation for two days at 25°C, 30°C or 37°C. Yeast strains containing deletions or mutation of *SSA1-4* and *SSE1* genes were generous gifts from Dr. Elizabeth Craig (University of Wisconsin-Madison), and Kevin Morano (University of Texas Medical School at Houston), respectively. Radicicol was obtained from Sigma.

**Table 1:** Strains used in this study.

Strain		Genotype	Source or reference
**WT ** ***UTP21***			
JJ762	*MAT* **a**	*ade2-1 ura3-1 leu2-3,112 trp1-1 met2 his3-11,15 lys2*	E.A. Craig
JJ623	*MAT* **a**	*sti1::MET*	[57]
JJ665	*MATα*	*hsc82::LEU2*	This study
JJ160	*MAT* **a**	*ydj1::HIS3 [pRS316-YDJ1]*	[27]
JJ613	*MAT* **a**	*ssa1-45:URA3 ssa2::TRP1 ssa3::LYS3 ssa4::ADE2*	This study
JJ517	*MAT* **a**	*sse1::kan^r^*	This study
JJ816	*MAT* **a**	*hsp82::LEU2 hsc82::LEU2/YEp24-HSP82*	[57]
***utp21-S602F***			
JJ59	*MAT* **a**	*ade2-1 ade3 MET2 sti1::TRP utp21-S602F [pRS316-ADE3STI1]*	[5]
JJ606	*MATα*	*utp21-S602F MET2*	This study
JJ549	*MATα*	*utp21-S602F sti1::MET2 [pRS316-ADE3STI1]*	This study
JJ605	*MATα*	*utp21-S602F hsc82::LEU2*	This study
JJ536	*MATα*	*utp21-S602F ydj1::HIS3 [pRS316-YDJ1]*	This study
JJ708	*MATα*	*ssa1-45:URA3 ssa2::TRP1 ssa4::ADE2 utp21-S602F*	This study
JJ809	*MAT* **a**	*sse1::kan^r^ utp21-S602F*	This study
JJ444	*MAT* **a**	*ade2-1 ade3 aha1::kan^r^ utp21-S602F [pRS316-ADE3AHA1]*	This study
JJ442	*MAT* **a**	*ade2-1 ade3 hch1::kan^r^ utp21-S602F [pRS316-ADE3HCH1]*	This study
JJ461	*MATα*	*ade2-1 ade3 sba1::kan^r^ utp21-S602F [pRS316-ADE3SBA1]*	This study
JJ492	*MAT* **a**	*ade2-1 ade3 ppt1::kan^r^ utp21-S602F [pRS316-ADE3PPT1]*	This study
JJ476	*MAT* **a**	*ade2-1 ade3 LYS2 cpr6::kan^r^ utp21-S602F [pRS316-ADE3CPR6]*	This study
JJ533	*MATα*	*ade2-1 ade3 tah1::kan^r^ utp21-S602F [pRS316-ADE3TAH1]*	This study
JJ712	*MAT* **a**	*hsp82:LEU2 hsc82::LEU2 utp21-S602F/YEp24-HSP82*	This study
***utp21::MET2***			
JJ666	*MAT* **a**	*utp21::MET2 [YCp50-UTP21]*	This study
JJ600	*MAT* **a**	*sti1::MET2 upt21::MET2 [YCp50-UTP21]*	This study
JJ591	*MAT* **a**	*hsc82::LEU2 hsp82::LEU2 utp21::MET2 [pRS316-HSC82 UTP21]*	This study
JJ714	*MATα*	*ydj1::HIS3 utp21::MET2 [pRS315-YDJ1] [YCp50-UTP21]*	This study
JJ414	*MATα*	*ydj1::HIS3 utp21::MET2 [pRS315-ydj1-G315E] [YCp50-UTP21]*	This study
**GFP strains**			
JJ598	*MAT* **a**	*Utp21-GFP:HIS3*	This study
JJ717	*MAT* **a**	*Utp21-S602F-GFP:HIS3*	This study
JJ1024	*MAT* **a**	*Utp6-GFP:HIS3*	This study
JJ455	*MAT* **a**	*hsc82::LEU2 hsp82::LEU2 Utp21-GFP:HIS3 [YEp24-HSP82]*	This study

### UTP21 Plasmids and Construction of the *utp21::MET2 Strain*

JJ59 (*ade2 ade3 MET2 utp21-S602F sti1::TRP1/pRS316-ADE3STI1*) contains the *utp21-S602F* mutation [5]. Strain JJ59 was grown in the presence of 5-FOA (Toronto Chemicals), which negatively selects for the *pRS316-ADE3STI1* plasmid. The 37°C growth defect of those cells was rescued by a plasmid encoding *UTP21* (YCp50-*UTP21*). A genomic *SalI-SpeI* fragment containing the *UTP21* gene was cloned into pRS315 (pRS315-*UTP21*) [58]. The *utp21-S602F* mutation, which disrupts an *AciI* restriction enzyme site, was identified by gap repair followed by DNA sequencing. JJ606 (*utp21-S602F*) was constructed by crossing JJ59 to an otherwise WT strain and identifying slow growing strains that lost the specific *AciI* restriction site. The *utp21::MET2* disruption strain was made by insertion of the *MET2* gene into *BamH1-Pst1* sites of *UTP21*. JJ666 (*utp21::MET2*/YCp50-*UTP21*) dies in the presence of 5-FOA unless another copy of *UTP21* is supplied.

Plasmid-borne TAP-tagged versions of Utp21 were constructed from a commercially available *UTP21* clone containing a C-terminal tandem affinity tag expressed under the *GAL1* promoter (Open Biosystems) [59]. *Spe*I and *Xho*I sites were introduced to facilitate cloning of Utp21-TAP into vectors with varying levels of constitutive expression (413*GPD*, 413*TEF,* 413*ADH* and 414*ADH*) [30]. S602F or other amino acid alterations were introduced into these vectors using PCR-mutagenesis followed by confirmatory DNA sequencing.

### Additional yeast strains

To construct additional yeast strains, the appropriate gene disruption cassette (e.g. *ppt1::kan^r^*) was amplified out of the commercially available strain (Open Biosystems), transformed into JJ762, then crossed to strains containing the *utp21-S602F* mutation. A similar approach was used to obtain isogeneic strains expressing Utp21-GFP, Utp21-S602F-GFP or Utp6-GFP. Yeast strains expressing *Utp21-GFP:HIS3* or *Utp6-GFP:HIS3* from the endogenous chromosomal location were purchased from Invitrogen [40]. Oligonucleotide primers were used to amplify a cassette including the carboxy-terminus of Utp21 fused to GFP. The localization of Utp21-GFP in the W303 background was indistinguishable from that in the commercial strain (not shown). To obtain a strain with chromosomally expressed Utp21-S602F-GFP, the Utp21-GFP cassette was transformed into the JJ606 (*utp21-S602F*) strain. Standard techniques confirmed that WT or mutant *Utp21-GFP:HIS3* was allelic to *utp21::MET2*.

### Additional Co-chaperone Plasmids

WT or mutant His-tagged Hsc82 (Hsp90) was expressed from pRS313GPDHis-*HSC82* or the related pRS314GPDHis-*HSC82* plasmid. Untagged WT or mutant Hsc82 was expressed from the pRS313-*HSC82* plasmid [34]. pRS316-*HSC82UTP21* expresses WT genomic clones of both genes. *ADE3* was cloned into pRS316 as a 3.3 kb *Bst1107/Nhe1* fragment. Co-chaperones were cloned into pRS316-*ADE3* using the indicated engineered restriction sites: *HCH1* (*SacI-BamHI*), *SBA1* (*SacI-XbaI*), and *AHA1* (*NotI-BamHI)*. *pRS316ADE3-CPR6*, pRS316*ADE3-PPT1* and pRS316*ADE3-TAH1* have been described [60]. WT *YDJ1* or *ydj1-G315E* were expressed from the pRS315 plasmid [27].

### Isolation of Utp21 Complexes and Immunoblot Analysis

To isolate Utp21-TAP complexes, *utp21::MET2* cells expressing pRS414*ADHUTP21* were grown overnight to an OD._600_ of ∼2.0. Cells were disrupted in lysis buffer (20 mM Tris, pH 7.5, 100 mM KCl, 5 mM MgCl_2,_ plus a protease inhibitor tablet (Roche Applied Science)) in the presence of glass beads. Yeast lysate was incubated with IgG sepharose (Amersham Biosciences) (1 hour, 4°C) followed by washes with 20 mM Tris-HCl pH 7.5, 100 mM KCl, 5 mM MgCl_2_, 0.1% Tween-20. Proteins bound to IgG sepharose were eluted by boiling in SDS-PAGE sample buffer and separated by gel electrophoresis (7.5% SDS-polyacrylamide gel unless otherwise indicated). Alternatively, cells (0.5 OD._600_ units) were resuspended in cold phosphate-buffered saline containing 1 mM phenylmethylsulfonylfluoride and disrupted with glass beads in the presence of SDS and triton X-100. For immunoblot analysis, proteins were transferred to nitrocellulose and probed with indicated antibodies. Chemiluminescence immunoblots were performed according to the manufacturer’s suggestions (Pierce, Rockford, IL). Polyclonal anti-TAP antibodies were obtained from ThermoScientific. Polyclonal antibodies against Hsc82/Hsp82, Sti1 or Cpr6 have been described [34], [57]. The antibody against Zuo1 was a gift from Dr. Elizabeth Craig (University of Wisconsin-Madison).

### Utp21 Localization

Strains expressing GFP-tagged versions of Utp21 or Utp6 were grown overnight in selective media containing 10 μg/ml DAPI (4′,6-diamidino-2-phenylindole) and washed twice with water. Phase and immunofluorescence microscopy was performed on unfixed cells using a Nikon Eclipse 80i or Nikon Eclipse 1000 microscope and a 60×oil immersion objective. Fluorescent images were deconvolved using NIS-Elements AR 3.0 or MetaMorph software, respectively.
